# Support staff’s perceptions of discontinuing antipsychotics in people with intellectual disabilities in residential care: A mixed‐method study

**DOI:** 10.1111/jar.12577

**Published:** 2019-02-20

**Authors:** Bas Kleijwegt, Addy Pruijssers, Lydie de Jong‐Bakker, Koos de Haan, Harmieke van Os‐Medendorp, Berno van Meijel

**Affiliations:** ^1^ Esdégé‐Reigersdaal Heerhugowaard The Netherlands; ^2^ Department of Nursing Science University Medical Centre Utrecht Utrecht The Netherlands; ^3^ Inholland University of Applied Sciences Amsterdam The Netherlands; ^4^ De Rotonde, Centre of Expertise ID‐MH Heerhugowaard The Netherlands; ^5^ School of Health Care Zwolle The Netherlands; ^6^ Department of Dermatology and Allergology University Medical Centre Utrecht The Netherlands; ^7^ Clinical Health Sciences University Medical Centre Utrecht The Netherlands; ^8^ Department of Psychiatry, Amsterdam Public Health Research Institute VU University Medical Center Amsterdam The Netherlands; ^9^ Parnassia Psychiatric Centre The Hague The Netherlands; ^10^ GGZ-VS Academy for Masters in Advanced Nursing Practice Utrecht The Netherlands

**Keywords:** antipsychotics, challenging behaviour, intellectual disabilities, mixed methods, perceptions

## Abstract

**Background:**

Although there is little evidence on their efficacy regarding challenging behaviour, antipsychotics are the most used psychotropic drugs in residential intellectually disabled people. Discontinuation is possible for some residential clients with intellectual disabilities. This study aimed to gain insight into support staff's perceptions of discontinuing antipsychotics in residential clients with intellectual disabilities.

**Method:**

Four focus groups were conducted in this mixed‐methods study, followed by a survey.

**Results:**

A large majority of support staff perceive antipsychotics to be effective in controlling challenging behaviour. Support staff regarded themselves as willing to contribute to the discontinuation of antipsychotics, but were more confident about achieving reductions.

**Conclusions:**

The attitude of the majority of support staff towards discontinuation provides a good basis for regularly reviewing antipsychotics use. A reduction plan should include preliminary steps, methods of monitoring and evaluating the process, and establishing measures for dealing with possible crises.

## 1 INTRODUCTION

In residential facilities, antipsychotics are the most commonly used psychotropic drugs in people with intellectual disabilities (de Kuijper et al., 2010; Deb et al., 2009; Stolker, Koedoot, Heerdink, Leufkens, & Nolen, 2002). In the Netherlands, the estimated prevalence of their use in residential care in these adults is 32%; in 2010, it was found that 78% of them had been using antipsychotics for over ten years (de Kuijper et al., 2010).

In people with ID, antipsychotics are used for registered indications such as schizophrenia and psychotic episodes, but also for behavioural symptoms without psychotic components, such as aggression or self‐injurious behaviour (so‐called “off‐label” use) (De Kuijper, Van Loon, Steegemans, & Ewals, 2007).

A recent study showed that 58% of residents with intellectual disabilities on antipsychotics used this type of medication for behavioural problems (de Kuijper et al., 2010). These behavioural problems are also referred to as challenging behaviour (CB) without psychotic components. CB is defined as “culturally abnormal behaviour(s) of such intensity, frequency or duration that the physical safety of the person or others is likely to be placed in serious jeopardy; or behaviour which is likely to seriously limit use of, or result in the person being denied access to, ordinary community facilities” (Emerson, 1995). Although antipsychotics are frequently prescribed for people with intellectual disabilities and CB, their efficacy is questioned. A systematic review by Matson and Neal, based on twelve studies, showed no unambiguous positive effects of antipsychotic medication on CB in adults with intellectual disabilities (Matson & Neal, 2009). Eight studies showed a significant reduction of CB, whereas four studies did not demonstrate such effects. A number of studies among children with autism and/or intellectual disabilities showed the superiority of risperidone over placebo in RCTs but the findings among adults with intellectual disabilities are not conclusive (Deb, 2016). A major disadvantage of antipsychotics is that it can cause serious neurological, metabolic, cardiovascular, haematological, gastro‐intestinal and genito‐urinary side effects (Cahn et al., 2008; de Kuijper et al., 2010; de Leon, Greenlee, Barber, Sabaawi, & Singh, 2009).

Given not only the limited evidence on the efficacy of antipsychotic medication for CB in people with ID, but also the occurrence of sometimes serious side effects, the options for its discontinuation are debated in the scientific literature. The first systematic review on reducing or completely discontinuing these antipsychotics in this group found that it is possible to reduce or discontinue them in a substantial number of clients, although not always without adverse effects (Sheehan & Hassiotis, 2016). In the 21 studies in question, the proportion of clients in whom antipsychotics were discontinued ranged from 4% to 74%. The proportion in whom dose reduction was maintained at follow‐up ranged from 19% to 83%. The review concluded overall that, although antipsychotics can successfully be reduced or discontinued in a substantial proportion of clients, this is not appropriate in another subgroup due to the re‐occurrence of behavioural deterioration.

The same review also showed that no reliable predictors of poor response could be identified. The effects of discontinuation varied between the included studies (*n* = 21): after reduction or discontinuation, there was weight loss (*n* = 2), improvement of metabolic parameters (*n* = 1) and improvement of cognitive functioning (*n* = 2). Despite the intention of reducing side effects, dyskinesia increased, sometimes transiently (*n* = 8). Six of the studies reported behavioural outcomes related to discontinuation or reduction. In all six studies, a substantial subgroup was found which showed no (persistent) behavioural deterioration.

If antipsychotics are to be discontinued or reduced effectively, multidisciplinary collaboration is necessary, with the active involvement of the support staff—that is, those who provide daily care and support to people with ID. By observing and reporting behaviour in such clients, these professionals are crucial to proper decision making on the subject (Sheehan & Hassiotis, 2016). They are also the first to be confronted with the possible consequences of the discontinuation in terms of symptomatic deterioration or behavioural disturbances. Besides support staff, also psychologists and physicians will play an important role in clinical decision making regarding discontinuation.

Clinical experience has shown that the topic of reduction or discontinuation of psychotropic medication not only calls for restraint, but also raises concern and/or fear in support staff. However, little is known scientifically about support staff's perceptions on the matter. Although professionals’ perceptions on use of psychotropic medication in general in people with intellectual disabilities was investigated in some older studies (Aman, Singh, & White, 1987; Christian, Snycerski, Singh, & Poling, 1999; Singh et al., 1996) and in one more recent study (Lalor & Poulson, 2013), none of these studies focused specifically on perceptions on reduction or discontinuation of antipsychotics. Recently, a study was published about knowledge of psychotropic medication of support staff and expectations of support staff regarding the effects of antipsychotics on CB (De Kuijper & Putten, 2017). This study showed that a majority of support staff had expectations of antipsychotics having positive effects on behavioural and cognitive functioning of people with ID, and the researchers classified these expectations as unrealistic.

As support staff are central to care for people with ID, it is crucial to gain insight into their perceptions, which—positively and negatively—can significantly influence the discontinuation process. Greater scientific insight will therefore make it possible to discuss within a multidisciplinary context, possible strategies for successfully discontinuing or reducing these drugs in this population.

This mixed‐methods study was thus intended to gain insight into support staff's perceptions on reducing or discontinuing the long‐term use of antipsychotics in residential clients with ID.

## 2 METHODS

### 2.1 Design

To investigate support staff's perceptions regarding the discontinuation of antipsychotics in residential clients with ID, we conducted a mixed‐methods study (Creswell & Clark, 2011). Through focus group interviews, the first phase consisted of a qualitative exploration of their perceptions. As a method of qualitative data collection, the use of focus groups is an appropriate way of gaining insight into participants’ attitudes, perceptions and opinions (Krueger, 1994). As such a group setting offers opportunities for participants to discuss controversial issues, it has added value over individual interviews (Ketelaar, Hentenaar, & Kooter, 2011). Another strength of these interviews is that they make it possible to discuss matters extensively and to provide insights into the various perspectives of the focus group members.

Next, based on the prevalent perceptions about discontinuing antipsychotics we developed a questionnaire for use in a survey among support‐staff members to quantify these perceptions in a larger sample.

The epistemological stance for our mixed‐methods research is a pragmatic one, where the research question determined the successive stages in the research process, starting with qualitative research to obtain in‐depth insights into the research topic, and followed by quantitative research to measure variables and statistical trends (Creswell & Clark, 2011; Polit & Beck, 2012; Tashakkori & Teddlie, 2003).

### 2.2 Focus groups

To investigate support staff's perceptions, we conducted four focus groups at three organizations providing residential care for people with intellectual disabilities in the Netherlands, many of whom have severe ID. To meet our inclusion criteria, support‐staff members had to work on a ward where at least 30% of the clients used antipsychotics. Twenty‐two managers from these wards were asked to recruit support staff for these focus groups. The wards differed with respect to age of the client population, intensity of support and severity of ID. Eventually, with the active contribution of 11 managers, a convenience sample of 29 participants was composed. All participants received an information letter and signed an informed consent form.

To structure the focus group interviews, a topic list was compiled both on the basis of findings from the literature and of inputs by members of an expert group that was composed in preparation for this study. Using the search terms “intellectual disability,” “antipsychotics,” “perceptions,” and synonyms, the PsycInfo, CINAHL, ERIC and PubMed databases were used to find relevant literature. This produced several studies on the prevalence, efficacy and side effects of antipsychotics in people with ID. It also produced some discontinuation studies (Ahmed et al., 2000; De Kuijper, Evenhuis, Minderaa, & Hoekstra, 2014; May et al., 1995; Stevenson et al., 2004) and some older studies on support staff's general perceptions of the use of psychotropic medication (Aman et al., 1987; Christian et al., 1999; Singh et al., 1996). These all provided inputs for the formulation of topics for the focus group interviews.

The advisory expert group consisted of an intellectual disabilities physician, a ward manager, a senior researcher specialized in anxiety in people with ID, and a manager supervising medical staff, paramedical staff and psychologists. The expert group commented on a first draft of the topic list. Some items were removed and others added. This resulted in the following sequence of main topics: indications for the use of antipsychotics, reasons for discontinuation, attitude towards discontinuation and the preconditions for effective discontinuation (see Table 1).

**Table 1:** Topic list

**Indications for the use of antipsychotics**
In which cases or situations are antipsychotics used? How do they help (1) from the perspective of support staff and (2) from the perspective of clients with ID?
**Reasons for discontinuation**
What are the reasons for considering discontinuation of antipsychotics?
**Expectations regarding the discontinuation process**
How, in positive or negative terms, do you expect the discontinuation process to develop? What is this expectation based on?
**Attitude towards discontinuation**
What is your attitude towards the discontinuation of antipsychotics? Which factors influence it? What is the relative weight of each factor? Under which circumstances can discontinuation be considered either a success or a failure?
**Preconditions for discontinuation**
Which preconditions are necessary for discontinuation? How do they influence the discontinuation process? What are the relative weights of the preconditions?

All focus groups were moderated by the fourth author of this article (KH), an expert nurse and MSc in nursing, who is also an experienced moderator of focus groups. He has extensive experience of working with people with intellectual disabilities and CB.

The focus groups lasted approximately 1.5 hr, were audio‐recorded and were transcribed verbatim. Using the qualitative software program NVIVO10, a thematic analysis of the transcriptions was conducted according to Braun and Clarke (2006). To reach agreement on the relevant codewords, the first focus group interview was coded independently by two researchers (BK, LdJ). The next three focus group interviews were coded by the first author (BK), the results being discussed afterwards with the third author (LdJ), a registered nurse and student in nursing science who has extensive working experience with the target group. Relevant themes were determined by the first author and discussed with the third author. The final themes from the focus group interviews were then presented to the participants of the focus group interviews for review (member check). Nine participants responded to our invitation to review the selected themes. They all confirmed the content of our report with the elaboration of themes. The member check did not lead to any further adjustments of the identified themes.

### 2.3 Survey

On the basis of the findings from the focus group interviews, which referred to support staff's perceptions of the discontinuation of antipsychotics, an item pool was generated by the research team. Discussion of this with the expert group led to a first selection of items for the survey. The research team reviewed these items to check that all the items would cover all the themes identified, and that the items would be clearly worded. Next, a first draft of the survey was formulated. It consisted of 29 statements to be scored on a 5‐point Likert response format. Two open‐ended questions were added on barriers and motivators with respect to discontinuation. The feasibility of the questionnaire was tested using cognitive interviewing (Beatty & Willis, 2007) by five professionals who would not participate in the actual survey. They were asked to complete the questionnaire while additional verbal information was collected with respect to (a) comprehension of the question (question intent, meaning of terms); (b) retrieval from memory of relevant information (recall ability of information; recall strategy); (c) decision process (motivation, sensitivity); and (d) response process (mapping of the response) (Willis, 1999). A few phrases were reformulated because their meaning proved to be ambiguous. Descriptive statistics were used to summarize the results of the survey. Answers on the open‐ended questions were analysed using content analysis.

## 3 RESULTS

### 3.1 Focus groups

The focus group interviews took place between November 2014 and February 2015. Twenty‐nine support‐staff members participated in the interviews, whereof 22 were female. The number of participants per focus group ranged from three to nine. The mean age of the participating support‐staff members was 44 years (range 19 to 62, *SD *= 12.0); their mean working experience was 16 years (range 2 to 32, *SD *= 9.5). Sixteen participants had trained in social work, eight had trained in nursing, and five had a combination of the two.

Two major themes were found: (1) balance between CB versus side effects (in particular blunted affect) and (2) the need for a proactive plan.

1. Balance between managing CB versus the occurrence of side effects, in particular blunted affect

Focus group participants assume that antipsychotics are effective in managing behavioural problems. At the same time, use of antipsychotic medication leads to a number of side effects, with negative influences on the quality of life of clients. Blunted affect is perceived as the side effect with most impact. In the perception of support staff, these side effects are often caused by a too high dosage of antipsychotics. On the other hand, a number of clients do need antipsychotics because of the CB and the corresponding “mental distress” they experience. So, efforts for discontinuation of antipsychotics should be considered when the balance between efficacy of antipsychotics and side effects is disturbed, that is, when the negative influence of the side effects predominates.

- The need for a proactive plan

Some participants described negative experiences regarding dose reduction or discontinuation of antipsychotic medication, which could have been prevented by a more proactive approach. In clinical practice, challenging situations occur during discontinuation of antipsychotic medication that have not been anticipated, thus leading to ineffective and unsatisfactory solutions, with distress for both clients and professionals. Participants explicitly stated that a methodical plan would improve their self‐confidence in dealing with such challenging situations regarding dose reduction or discontinuation. Most of the participants were convinced that there is a lot of room for improvement by developing a proactive, methodical plan.

In the following sections, more detailed findings obtained from the focus group interviews and (partly) related to the two main themes are described on the basis of main topics of the topic list.

#### 3.1.1 Indications for the use of antipsychotics

When focus group participants described why clients used antipsychotics, they referred in particular to managing behavioural or emotional disturbances, and less to the treatment of psychiatric disorders. They perceived antipsychotics as helpful in reducing anxiety, agitation, compulsive behaviour and self‐injurious behaviour in people with ID. They also considered antipsychotics effective for preventing overstimulation and overreaction in people with ID.

#### 3.1.2 Reasons for discontinuation

On the basis of their perception that too many people with intellectual disabilities took a too high dosage of antipsychotics and therefore suffered severe side effects, focus group participants were generally willing to work towards lower dosages. For some participants, the assumption that all medications had adverse effects led to the overall assertion that “less medication is better". Serious behavioural deterioration was seen as the main reason for not further discontinuing antipsychotic medication. Severe medical problems, such as medication overdose or severe fall risk, were considered as definite indications for reducing or discontinuing antipsychotics.

There was another reason for considering discontinuation. In some people with ID, the initial indication for them to use antipsychotics was unknown. In view of the current absence of evident behavioural or emotional disturbances, there were now no clear indications that antipsychotics should be used.

With regard to the management of CB in people with ID, some focus groups participants were aware of the alleged inefficacy of antipsychotics. In the absence of psychotic disorders, this gave them a reason to consider discontinuation. Also they assumed that, as people with intellectual disabilities and CB grow older, there was a chance of CB decreasing gradually as a natural process. At a certain point, it was therefore appropriate to consider discontinuation.

Finally, some participants referred to the trend in health care towards a reduction in the use of various coercive measures, such as fixative straps. In their view, the off‐label use of antipsychotics was also a coercive measure, which should thus be avoided as much as possible.

#### 3.1.3 Attitude towards discontinuation

Focus group participants formulated their attitudes towards discontinuation mainly on the basis of their previous experiences. They were convinced that it would be possible in very few cases both to discontinue antipsychotic medication completely without behavioural deterioration. In most cases, however, they felt that the main option was not to discontinue these drugs completely, but only to reduce them substantially, and that the limit would be determined by any significant increases in the symptoms of CB, such as self‐injurious behaviour or aggression. In unstable people with intellectual disabilities with persistent CB, even a reduction of antipsychotics was not considered appropriate.

Some participants stated explicitly that they expected a temporary increase in CB during the period of withdrawal. Anticipating severe, irreversible behavioural deterioration, even after a return to the original dose of antipsychotics, some participants saw any discontinuation of antipsychotics as a significant risk to people with ID. In their view, this was an insurmountable barrier against any experiments intended to discontinue antipsychotics.

#### 3.1.4 Preconditions for discontinuation

The participants saw an environment with many unpredictable or threatening stimuli as unfavourable to the discontinuation of antipsychotics. A specific example was mentioned in which the presence of too many support‐staff members at a facility made it too difficult for them to approach a client in a predictable and safe manner. Also, if support staff have too little time to spend with an intellectual disabilities client who is eligible for discontinuation of antipsychotics, it will not be possible to provide enough support in managing stressful events and social interactions, and in managing any temporary or long‐term exacerbations of CB.

In the participants’ view, anxiety and aggression would be reduced by a positive approach that focused on support rather than control. This would increase the options for discontinuing antipsychotics. However, they had reservations about clients with a history of serious aggression incidents and with insufficient opportunities to control their disruptive behaviour. Even a strong supporting environment would then be insufficient to prevent and/or manage the disruptive behaviour of these clients.

Some participants explicitly indicated the need for a thorough plan for the discontinuation of antipsychotics, partly to prepare effectively for possible complications during the discontinuation process. In their view, such a plan should first formulate the positive outcomes that can be achieved by discontinuing or reducing antipsychotics. Second, it should also be determined whether there are any options for further optimizing the conditions for effective discontinuation, such as stress reduction, strengthening of client's coping skills, or the diagnosis and treatment of physical problems that contribute to CB. Thirdly, before discontinuation procedures start, it should be established whether support staff will be able to handle any temporary or longer‐term deterioration in a client's behaviour.

Participants stated that they would also find it helpful not only if different scenarios were developed regarding clients’ possible responses to the discontinuation of antipsychotics, but also if there were a description of the possible intervention strategies they could use if a client's behaviour deteriorated. Such interventions could be carried out in collaboration with other members of the multidisciplinary team, and with or without the help of outside experts on CB and crisis management. As crisis‐management experts have a more detached perspective of the client situation, they could help support staff overcome the negative emotions caused by any increase in CB (such as anger or fear). It would also be helpful to have clear agreement on the use of emergency medication.

It was also stated that it should be clear which person in the support‐staff team was responsible for communication with other members of the multidisciplinary team, especially the physicians. If communication were unstructured, ad hoc decisions could be made too easily, increasing the risk that more sceptical or fearful support‐staff members stopped or reversed the discontinuation process. Participants felt that decisions on (further) reduction or discontinuation could be based on the scores of a client's personal early recognition plan, indicating the severity of deterioration of symptoms and CB.

With respect to multidisciplinary collaboration, participants saw the physician as the leading professional in case of discontinuation of antipsychotics. Psychologists’ role in the discontinuation process was described less explicitly.

Noting that the opinion of relatives, as a client's legal representatives, weighed heavily in decisions on whether or not to reduce antipsychotics, for example by withholding their consent for the reduction of antipsychotics, the participants felt that the role and position of support staff was to some extent comparable with that of relatives. This was due to the relatives’ possibilities for monitoring the clients’ concrete behaviours (e.g., aggression, self‐injurious behaviour or more social behaviour) and for assessing the positive and negative consequences of discontinuing antipsychotics. They stated that this central position was not always acknowledged by the other professionals in the multidisciplinary team.

Finally, some participants stated that as they did not have sufficient knowledge about the positive and negative effects of discontinuing antipsychotics, they felt insecure about their role in the decision‐making process.

### 3.2 Survey

#### 3.2.1 Data collection and survey‐respondents’ characteristics

Data collection took place between April 2015 and June 2015. In total, 347 surveys were distributed in three organizations providing residential care to people with intellectual disabilities on wards where at least 30% of the clients used antipsychotics. A total of 187 surveys were returned (response rate 54%). The respondents’ mean age was 39 years (range 19 to 63, *SD *= 11.8); their mean working experience was 13 years (range 0 to 44, *SD *= 10.0). Sixty‐eight per cent of the respondents were female. Twenty per cent had had nursing training, 62% had trained in social work, and 14% had a combination of the two. The training background of 4% of the participants was unknown. The respondents’ characteristics are presented in Table 2.

**Table 2:** Characteristics of survey respondents

Number of respondents	187	
Mean age in years (*SD*/range)	38 (11.8/19–63)	
Years of working experience (*SD*/range)	13 (10.0/0–44)	
Percentage female/male/unknown	68/30/2	
Percentage with nursing training/social work training/nursing and social work training/unknown	20/62/14/4	

#### 3.2.2 Findings of the survey

Based on the topic list of the focus groups, the findings of the survey are presented in four clusters: (1) indications for the use of antipsychotics, (2) reasons for discontinuation, (3) attitude towards discontinuation and (4) the preconditions for discontinuation. In our presentation of these findings, the Likert scale answers “strongly disagree” and “disagree” were merged, as were the answers “agree” and “strongly agree.” The survey findings and the answers to the open‐ended questions are given below. A complete summary of the answers of the survey is presented in Table 3.

**Table 3:** Agreement with survey statements (expressed as a percentage)

	Strongly disagree	Disagree	Neither agree nor disagree	Agree	Strongly agree
Indications for the use of antipsychotics *n *(%)					
I know whether or not clients using antipsychotics have been diagnosed with a psychotic disorder.	0 (0)	13 (7)	35 (19)	120 (65)	17 (9)
Reasons for discontinuation of antipsychotics *n *(%)					
The side‐effects of antipsychotics are very stressful for clients.	0 (0)	9 (5)	89 (48)	82 (44)	7 (3)
Antipsychotics are effective in controlling challenging behaviour.	2 (1)	17 (9)	50 (27)	112 (60)	5 (3)
A considerable number of clients^a^ use antipsychotics but derive nearly no benefit from them.	5 (3)	63 (34)	76 (41)	40 (21)	1 (1)
For a considerable number^a^ of clients, the dosage of antipsychotics is too high.	2 (1)	37 (20)	86 (47)	56 (31)	2 (1)
Attitude towards discontinuation of antipsychotics *n *(%)					
I am ready and willing to contribute to discontinuing antipsychotics in some clients.	3 (2)	32 (17)	32 (17)	103 (55)	16 (9)
My team is too reserved about discontinuing antipsychotics.	15 (8)	62 (34)	66 (37)	37 (20)	2 (1)
Antipsychotics can be discontinued in a considerable number^a^ of clients.	7 (4)	69 (37)	68 (36)	40 (22)	2 (1)
Antipsychotics can be discontinued only in exceptional cases.	2 (1)	54 (29)	41 (22)	80 (43)	10 (5)
More clients should be prescribed antipsychotics.	13 (7)	102 (54)	63 (34)	7 (4)	1 (1)
Discontinuation is not possible at my place of work.	10 (5)	50 (27)	43 (23)	71 (38)	12 (7)
The dosage of antipsychotics can be decreased in a considerable number^a^ of clients.	1 (1)	16 (9)	42 (22)	115 (61)	13 (7)
Discontinuation of antipsychotics is a huge risk for clients.	3 (2)	35 (19)	98 (52)	47 (25)	4 (2)
Reducing antipsychotics almost always causes behaviour to deteriorate.	1 (1)	64 (34)	70 (37)	48 (26)	4 (2)
Reducing antipsychotics reduces safety more than can be justified.	2 (1)	60 (32)	82 (44)	38 (21)	4 (2)
Preconditions for discontinuing antipsychotics *n *(%)					
Sufficient account is taken of my input regarding the discontinuation of antipsychotics.	4 (2)	28 (15)	50 (27)	94 (52)	7 (4)
If there are safety issues, I can influence decisions on stopping or prolonging the discontinuation of antipsychotics.	7 (4)	32 (17)	29 (16)	108 (59)	8 (4)
If clients’ QoL is affected, I can influence decisions on stopping or prolonging the discontinuation of antipsychotics.	5 (3)	29 (16)	33 (18)	108 (58)	9 (5)
Decisions on discontinuation are taken too unilaterally by a physician.	11 (6)	101 (54)	46 (25)	20 (11)	7 (4)
The psychologist's expertise is indispensable in the process of discontinuing antipsychotics.	0 (0)	7 (4)	18 (10)	126 (68)	33 (18)
As a team of support staff, we need consultation from outside the team on discontinuing antipsychotics.	2 (1)	24 (13)	44 (24)	98 (54)	15 (8)
The opinion of a client's relatives should weigh heavily in decisions on discontinuing antipsychotics.	0 (0)	24 (13)	79 (43)	74 (41)	6 (3)
Discontinuing antipsychotics is possible only with a clear plan and a clear description of responsibilities.	0 (0)	1 (1)	8 (4)	126 (69)	49 (26)
Knowing how a client can react to discontinuation of antipsychotics is supportive in guiding clients.	0 (0)	3 (2)	8 (4)	140 (76)	33 (18)
My knowledge is sufficient to allow me to take part in multidisciplinary deliberations on the discontinuation of antipsychotics.	3 (2)	52 (28)	50 (27)	76 (41)	4 (2)
I recognize the side‐effects of antipsychotics.	0 (0)	17 (9)	53 (29)	112 (60)	4 (2)
I am aware of the possible harmful long‐term side‐effects of antipsychotics.	0 (0)	29 (15)	42 (23)	103 (56)	11 (6)
I know when to consult a physician about side‐effects.	0 (0)	11 (6)	28 (15)	138 (75)	8 (4)
I am aware of the phenomena that may occur during discontinuation of antipsychotics.	0 (0)	18 (10)	24 (13)	135 (73)	8 (4)

Notes

QoL: quality of life.

a “a considerable number of clients” was defined as “a quarter or more”.

##### Indications for the use of antipsychotics

*Off‐label use*: The first statement referred to the support staffs’ knowledge of whether the clients using antipsychotics had been diagnosed with a psychotic disorder. A large majority of respondents (74%) confirmed that they knew whether or not clients using antipsychotics have been diagnosed with a psychotic disorder.

##### Reasons for discontinuation

*Side effects*: The survey showed that 47% of respondents perceived the side effects of antipsychotics to be very stressful for clients, and that only 5% disagreed that this was the case. Almost half the respondents (48%) neither agreed nor disagreed.

*Efficacy*: The efficacy of antipsychotics was barely questioned: only 10% of respondents disagreed with the statement that antipsychotics are effective in controlling CB. At the same time, 22% agreed with the statement that even though they have almost no benefit, antipsychotics are used for a considerable number of the clients.

*Too high a dosage*: Thirty‐two per cent of respondents stated that the dosage of antipsychotics was too high in a considerable number of clients. Twenty‐two per cent disagreed. A large number of respondents (47%) neither agreed nor disagreed.

##### Attitude towards discontinuation

*Motivation*: A majority of support staff (64%) regarded themselves as willing to contribute to the discontinuation of antipsychotics in a proportion of their clients. At team level, a minority (21%) considered their support‐staff team to be too reluctant about discontinuing antipsychotics.

*Possible success rate*: Almost half of the respondents (41%) felt that antipsychotics could not be discontinued in a considerable number of clients, while 23% felt that they could be discontinued. This is in line with the finding that 45% of respondents believed that discontinuation of antipsychotics would not be possible at their own facility. Forty‐eight per cent of respondents agreed with the statement that complete discontinuation can be reached only in exceptional cases, while 22% neither agreed nor disagreed. This left a minority of 30% with a more positive expectation regarding the number of clients in whom complete discontinuation was possible. However, a 68% majority of respondents felt that it would be possible to reduce the dosage in a considerable number of their clients. Only 5% of respondents agreed with the statement that more clients should be prescribed antipsychotics.

While 52% neither agreed nor disagreed with the statement that discontinuation is a huge risk for clients, comparable numbers of respondents agreed (27%) and disagreed (21%).

*Consequences of discontinuation*: While a 28% minority of respondents felt that reducing antipsychotics always caused a behavioural deterioration in clients, 35% disagreed with this statement. And while 23% of respondents agreed with the statement that the discontinuation of antipsychotics causes an unjustifiable lack of safety, 33% disagreed.

##### Preconditions for discontinuation

*Influence*: A 56% majority of respondents perceived that sufficient account was taken of their input within the multidisciplinary team regarding decisions on discontinuing or reducing antipsychotics, while 17% considered that it was not. Almost two‐thirds of respondents felt they could influence decisions about discontinuation in relation to safety issues (63%) or a client's quality of life of (63%). While 15% believed that decisions about discontinuation were taken too unilaterally by a physician, 60% disagreed. A quarter was thus undecided.

*Multidisciplinary collaboration*: A large majority (86%) perceived the psychologist's expertise on discontinuing antipsychotics to be indispensable. A large number of respondents (62%) considered consultation from outside the team to be necessary to discontinuing antipsychotics in a responsible manner. While almost half the respondents (44%) considered that relatives’ opinions on decisions to discontinue were important, 13% disagreed, and 43% were undecided.

*Plan*: Almost full agreement was reached on the statement that discontinuation needs a clear plan. Respondents felt that having different scenarios how clients could react would help them to anticipate clients’ possible reactions during discontinuation or dosage reduction.

*Knowledge*: In their perception, a large number of respondents had sufficient knowledge for the following: taking part in multidisciplinary consultation (43%), being able to recognize side effects of antipsychotics (62%) and being aware of the possible harmful long‐term side effects of antipsychotics (62%). Seventy‐nine per cent perceived themselves as being able to determine when to consult a physician in the event of serious side effects, and 77% felt that they were aware of possible occurrence of phenomena or withdrawal symptoms during discontinuation.

##### Open‐ended questions

In the open‐ended questions, respondents were asked what motivated them most about contributing to the discontinuation of antipsychotics, and what were important barriers to doing so. A number of 161 respondents answered the question about motivating factors, and 140 respondents answered the question about barriers.

Six motivating factors were identified: reducing side effects (*n* = 44, 24%), decrease of blunted affect (*n* = 24, 13%), belief in the general principle of “the less medication, the better” (*n* = 19, 10%), a belief that the efficacy of antipsychotics diminished over time (*n* = 9, 6%), the need to reduce too high a dosage of antipsychotics (*n* = 7, 4%) and the options for providing tailored support rather than behavioural management through antipsychotics (*n* = 6, 3%).

Nine barriers were identified: the risk that behavioural deterioration would recur, threatening the safety either of a client (*n* = 12, 6%) or of support staff (*n* = 12, 6%); a belief that support staff had insufficient influence on the discontinuation process (*n* = 11, 6%), a belief that respondents had insufficient knowledge (*n* = 11, 6%), uncertainty due to the expected unpredictability of the clients’ behaviour that would result from discontinuation (*n* = 9, 5%), lack of sufficient time to support clients (*n* = 8, 5%), insufficient availability of multidisciplinary consultation (*n* = 7, 4%), insufficient competencies of team members to cope with possible behavioural deterioration (*n* = 7, 4%) and the danger that the dose of antipsychotics would be reduced too fast (*n* = 5, 3%).

## 4 DISCUSSION

In this mixed‐methods study, four focus groups preceded a survey intended to gain insight into support staff's perceptions on discontinuing antipsychotics in residential clients with ID.

A large majority of support staff were of the opinion that antipsychotics control CB effectively. They were aware of the side effects of antipsychotics and viewed blunted affect as the most stressful side effect. Although a majority of support staff considered themselves to be willing to contribute to the discontinuation of antipsychotics, they were more confident about achieving dose reductions than about achieving complete discontinuation. They underscored the importance of a number of conditions regarding the successful reduction or complete discontinuation of antipsychotics, for example availability of sufficient time to support the client during stressful events, the availability of a plan to respond to possible complications during discontinuation and structured communication with other members of the multidisciplinary team.

When comparing the outcomes of this current study with the study of expectations of support staff regarding the effects of antipsychotics (De Kuijper & Putten, 2017), both studies found that a majority of support staff considered antipsychotics as effective in controlling or diminishing challenging behaviour. The positive view of support staff is not supported by unambiguous scientific evidence on the antipsychotics’ efficacy in controlling CB (Deb, 2016; Matson & Neal, 2009).

Given the absence of reliable predictors for successful discontinuation, a methodically supported trial‐and‐error strategy seems to be the only way to determine a specific individual's options for reduction or complete discontinuation (Sheehan & Hassiotis, 2016). Our study showed that, when discontinuing antipsychotics, support staff regarded a thorough, tailor‐made and proactive plan to be supportive. As well as preparing, monitoring and evaluating the discontinuation process, such a plan should include measures to improve support staff's capacity for dealing effectively with possible deterioration or crises. Based on the findings of our study and in line with Deb et al. (2009), we can make the following recommendations:

It is necessary to identify factors from the past that may trigger CB in the client. Next, proven effective strategies should be established that allow support staff and other members of the multidisciplinary team to prevent these triggers or to deal with them effectively. These strategies should provide options for emergency medication and practical assistance in the event of severe behavioural deterioration. When determining these strategies, it should also be determined whether specific conditions should be created that enable the support staff team to cope with any deterioration. Such conditions might include replacing temporary workers with permanent support staff or adding more experienced support staff.

In multidisciplinary collaboration, it will be necessary to develop a structured and tailored strategy for monitoring during discontinuation. This should focus on clients’ behaviour and quality of life, and also on the safety of clients and the people in their environment. As a reference point during the discontinuation process, a baseline assessment of the client's behaviour must also be established before discontinuation is started. This should include components such as aggressive behaviour, self‐injurious behaviour, agitation, restlessness, sleeping behaviour, eating behaviour, expressions of relaxation or joy, and other unique personal behaviours.

After this baseline assessment, the discontinuation procedures can start, and be followed by planned multidisciplinary evaluations, in which it will be necessary to decide on the pace of the subsequent steps and the phasing of dose reduction. In case of unforeseen events or crises, interim and unscheduled consultations should be organized. By contributing to the baseline behaviour assessment, the psychologist plays an important role in the preparatory phase, and also in the monitoring and evaluation phases, hypothesizing—in close collaboration with support staff—on possible explanations of changes in behaviour and determining appropriate intervention strategies.

Results from focus group interviews showed that most participants saw the physician as the leading professional in discontinuation of antipsychotics and described the psychologists role less explicitly. This finding is in line with the outcomes of a cost‐effectiveness study for the management of aggressive behaviour of Unwin and colleagues, who concluded that there is an overreliance on medication and psychiatrists, and a relatively lesser involvement from other multidisciplinary team members such as nurses and clinical psychologists (Unwin, Deb, & Deb, 2017). This was not reflected in the results of our survey item regarding the required psychologists expertise in the process of discontinuation of antipsychotics. More insights are needed on the division of tasks of physicians and psychologists regarding discontinuation of antipsychotics.

If antipsychotics reduction or discontinuation is to proceed according to the proposed plan, this generates a systematic approach to determine whether clients should indeed continue to take antipsychotics. If undertaken in a spirit of close multidisciplinary collaboration, a proactive, cautious, careful and methodical approach to such a plan will also enhance support staff's professional competence.

Strength of this study is that it is the first study to examine support staff's perceptions of discontinuation of antipsychotics in people with ID. Such staff play an important role in any discontinuation or reduction of antipsychotics: they are the first to be confronted with—and deal with—any changes in behaviour, and the first to observe and report relevant signs and symptoms in these clients. Another strength of this study lies in the content of the survey, which, to list relevant perceptions about the discontinuation of antipsychotics, was based on insights gained not only from the available literature, but also from preparatory focus groups. Another strength is that, through the focus groups and survey, we collected data in three different organizations. This increases the generalizability of our findings. This study was conducted in a Dutch residential setting, whose care facilities may have differed from those in other countries. It nonetheless seems reasonable to assume that support staff in other settings, including ambulatory settings, face similar challenges regarding reducing or discontinuing antipsychotics.

The most important limitation of this study is that this study was conducted in residential clients with ID, most of them with severe ID. This means that staff's perceptions regarding people with a mild intellectual disabilities are underexposed.

## 5 CONCLUSION

A majority of support staff considers antipsychotics effective in controlling CB. This position is not supported by unambiguous scientific evidence. Despite their limited confidence in the feasibility of discontinuing antipsychotics, we conclude that most support staff considers themselves to be willing to collaborate on discontinuing them where possible. When considering or implementing discontinuation or reduction, a detailed plan is needed. Such a plan should develop strategies for effective monitoring and for regular evaluations during the discontinuation or reduction process. It should also include measures necessary to improving support staff's capacities for dealing effectively with possible crises.

To optimize discontinuation procedures, further research is needed on the development and evaluation of discontinuation interventions.

## References

[jar12577-bib-0001] Ahmed, Z. , Fraser, W. , Kerr, M. P. , Kiernan, C. , Emerson, E. , Robertson, J. , & Thomas, J. (2000). Reducing antipsychotic medication in people with a learning disability. The British Journal of Psychiatry, 176(1), 42 10.1192/bjp.176.1.42 10789325

[jar12577-bib-0002] Aman, M. G. , Singh, N. N. , & White, A. J. (1987). Caregiver perceptions of psychotropic medication in residential facilities. Research in Developmental Disabilities, 8(3), 449–465. 10.1016/0891-4222(87)90025-4 3671821

[jar12577-bib-0003] Beatty, P. , & Willis, G. B. (2007). Research synthesis; the practice of cognitive interviewing. Public Opinion Quarterly, 71(2), 287–311. 10.1093/poq/nfm006

[jar12577-bib-0004] Braun, V. , & Clarke, V. (2006). Using thematic analysis in psychology. Qualitative Research in Psychology, 3(1), 77–101.

[jar12577-bib-0005] Cahn, W. , Ramlal, D. , Bruggeman, L. , De Haan, L. , Scheepers, F. , Van Soest, M. , … Slooff, C. (2008). Prevention and treatment of somatic complications of the use of antipsychotics [Preventie en behandeling van somatische complicaties bij antipsychoticagebruik]. Journal for Psychiatry [tijdschrift Voor Psychiatrie], 50(9), 579. 18785105

[jar12577-bib-0006] Christian, L. , Snycerski, S. M. , Singh, N. N. , & Poling, A. (1999). Direct service staff and their perceptions of psychotropic medication in non‐institutional settings for people with intellectual disability. Journal of Intellectual Disability Research, 43(2), 88–93. 10.1046/j.1365-2788.1999.00182.x 10221788

[jar12577-bib-0007] Creswell, J. , & Clark, V. (2011). Designing and conducting mixed methods research (2nd ed.). Thousand Oaks, CA: SAGE Publications Inc.

[jar12577-bib-0008] de Kuijper, G. , Hoekstra, P. , Visser, F. , Scholte, F. A. , Penning, C. , & Evenhuis, H. (2010). Use of antipsychotic drugs in individuals with intellectual disability (ID) in the Netherlands: Prevalence and reasons for prescription. Journal of Intellectual Disability Research, 54(7), 659–667. 10.1111/j.1365-2788.2010.01275.x 20426795

[jar12577-bib-0009] de Kuijper, G. , & Van Der Putten, A. J. (2017). Knowledge and expectations of direct support professionals towards effects of psychotropic drug use in people with intellectual disabilities. Journal of Applied Research in Intellectual Disabilities, 30, 861–9. 10.1111/jar.12357 28467003

[jar12577-bib-0010] De Kuijper, G. , Van Loon, Y. , Steegemans, H. , & Ewals, F. (2007). Prescription of psychotropic medication to people with intellectual disability NVAVG Guideline. Retrieved from http://www.nvavg.nl/upload/standaarden/standaard--2008-01-psychofarmaca.pdf

[jar12577-bib-0011] De Kuijper, H. , Evenhuis, H. , Minderaa, R. B. , & Hoekstra, P. J. (2014). Effects of controlled discontinuation of long‐term used antipsychotics for behavioural symptoms in individuals with intellectual disability. Journal of Intellectual Disability Research, 58(1), 71 10.1111/j.1365-2788.2012.01631.x 23046144

[jar12577-bib-0012] de Leon, J. , Greenlee, B. , Barber, J. , Sabaawi, M. , & Singh, N. N. (2009). Practical guidelines for the use of new generation antipsychotic drugs (except Clozapine) in adult individuals with intellectual disabilities. Research in Developmental Disabilities: A Multidisciplinary Journal, 30(4), 613–669. 10.1016/j.ridd.2008.10.010 19084370

[jar12577-bib-0013] Deb, S. (2016). Psychopharmacology In Singh N. N. (Ed.), Handbook of Evidence‐Based Practices in Intellectual and Developmental Disabilities (pp. 347–381). Cham: Springer International Publishing 10.1007/978-3-319-26583-4

[jar12577-bib-0014] Deb, S. , Kwok, H. , Bertelli, M. , Salvador‐Carulla, L. , Bradley, E. , Torr, J. , & Barnhill, J. (2009). International guide to prescribing psychotropic medication for the management of problem behaviours in adults with intellectual disabilities. World Psychiatry, 8, 181–186. 10.1002/j.2051-5545.2009.tb00248.x 19812757 PMC2758582

[jar12577-bib-0015] Emerson, C. (1995). Challenging behaviour: Analysis and intervention in people with learning difficulties. Cambridge: Cambridge University Press.

[jar12577-bib-0016] Ketelaar, P. , Hentenaar, F. , & Kooter, M. (2011). Groups in focus [Groepen in focus] (Vol. 1st). The Hague: Boom Lemma.

[jar12577-bib-0017] Krueger, R. (1994). Focus groups: a practical guide for applied research. New Delhi: Sage Publications.

[jar12577-bib-0018] Lalor, J. , & Poulson, L. (2013). Psychotropic medications and adults with intellectual disabilities: Care staff perspectives. Advances in Mental Health & Intellectual Disabilities, 7(6), 333–345. 10.1108/AMHID-03-2013-0017

[jar12577-bib-0019] Matson, J. L. , & Neal, D. (2009). Psychotropic medication use for challenging behaviors in persons with intellectual disabilities: An overview. Research in Developmental Disabilities, 30(3), 572–586, 10.1016/j.ridd.2008.08.007 18845418

[jar12577-bib-0020] May, P. , London, E. B. , Zimmerman, T. , Thompson, R. , Mento, T. , & Spreat, S. (1995). A study of the clinical outcome of patients with profound mental retardation gradually withdrawn from chronic neuroleptic medication. Annals of Clinical Psychiatry, 7(4), 155–160. 10.3109/10401239509149620 8721888

[jar12577-bib-0021] Polit, D. F. , & Beck, C. T. . (2012). Overview of mixed methods research In Nursing research: generating and assessing evidence for nursing practice (9th ed., pp. 603–651). Philadelphia, PA: Lippincott, Williams & Wilkins.

[jar12577-bib-0022] Sheehan, R. , & Hassiotis, A. (2016). Reduction or discontinuation of antipsychotics for challenging behaviour in adults with intellectual disability: A systematic review. The Lancet Psychiatry, 4(3), 238–256. 27838214 10.1016/S2215-0366(16)30191-2

[jar12577-bib-0023] Singh, N. N. , Ellis, C. R. , Donatelli, L. S. , Williams, D. E. , Ricketts, R. W. , Goza, A. B. , … Singh, Y. N. (1996). Professionals’ perceptions of psychotropic medication in residential facilities for individuals with mental retardation. Journal of Intellectual Disability Research, 40(1), 861–7. 10.1111/j.1365-2788.1996.tb00596.x 8930051

[jar12577-bib-0024] Stevenson, C. , Rajan, L. , Reid, G. , Melville, C. , McGilp, R. , & Cooper, S. A. (2004). Withdrawal of antipsychotic drugs from adults with intellectual disabilities. Irish Journal of Psychological Medicine, 21(3), 85 10.1017/S0790966700008417 30308735

[jar12577-bib-0025] Stolker, J. J. , Koedoot, P. J. , Heerdink, E. R. , Leufkens, H. G. M. , & Nolen, W. A. (2002). Psychotropic drug use in intellectually disabled group‐home residents with behavioural problems. Pharmacopsychiatry, 35(1), 19–23. 10.1055/s-2002-19838 11819154

[jar12577-bib-0026] Tashakkori, A. , & Teddlie, C. (2003). Handbook of mixed methods in social and behavioral research. Thousand Oaks, CA: Sage.

[jar12577-bib-0027] Unwin, G. , Deb, S. , & Deb, T. (2017). An exploration of costs of community‐based specialist health service provision for the management of aggressive behaviour in adults with intellectual disabilities. Journal of Applied Research in Intellectual Disabilities, 30(2), 316–325. 10.1111/jar.12241 26970410

[jar12577-bib-0028] Willis, G. B. (1999). Cognitive Interviewing, A “How To” guide. http://www.chime.ucla.edu/publications/docs/cognitive%20interviewing%20guide.pdf

